# Editorial: Ovarian cancer targeted medication: PARP inhibitors, anti-angiogenic drugs, immunotherapy, and more, volume II

**DOI:** 10.3389/fphar.2025.1552652

**Published:** 2025-02-10

**Authors:** Zhi-Bin Wang, De-Hua Liao, Guang Lei, Zhao-Qian Liu, Nayiyuan Wu, Jing Wang

**Affiliations:** ^1^ Hunan Key Laboratory of Cancer Metabolism, Central South University/Hunan Cancer Hospital, Changsha, Hunan, China; ^2^ Public Service Platform of Tumor Organoids Technology, Hunan Gynecological Tumor Clinical Research Center, Changsha, Hunan, China; ^3^ Department of Pharmacy, The Affiliated Cancer Hospital of Xiangya School of Medicine, Central South University/Hunan Cancer Hospital, Changsha, China; ^4^ Department of Experimental Radiation Oncology, The University of Texas MD Anderson Cancer Center, Houston, TX, United States; ^5^ Hunan Key Laboratory of Pharmacogenetics, Department of Clinical Pharmacology, National Clinical Research Center for Geriatric Disorders, Xiangya Hospital, Central South University, Changsha, Hunan, China; ^6^ Institute of Clinical Pharmacology, Engineering Research Center for Applied Technology of Pharmacogenomics of Ministry of Education, Central South University, Changsha, Hunan, China

**Keywords:** ovarian cancer, targeted medication, immunomodulatory, drug resistance, PARP inhibitors, anti-angiogenic drugs

Ovarian cancer (OC), the deadliest gynecological malignancy, primarily relies on tumor debulking and post-surgical platinum-based chemotherapy. However, platinum resistance often emerges after multiple recurrences. Given OC’s heterogeneity and complex molecular landscape, pinpointing specific molecular targets is crucial for understanding its mechanisms and progression. The therapeutic paradigm for OC is evolving from traditional chemotherapy to targeted therapies, with PARP inhibitors and anti-angiogenic agents becoming key maintenance treatments. Despite their promise, these therapies face challenges such as inefficacy, adverse effects, and cost. Next-generation sequencing (NGS) offers a broader spectrum of targeted agents, potentially enhancing personalized treatment strategies. Additionally, immunotherapy and ferroptosis modulation present innovative avenues for OC treatment. Enhancing the efficacy and reducing the side effects of current OC drugs, as well as exploring new targets, are pressing needs. We seek to identify novel therapeutic targets and biomarkers for OC, encouraging both computational and experimental pharmacological studies.

**FIGURE 1:**
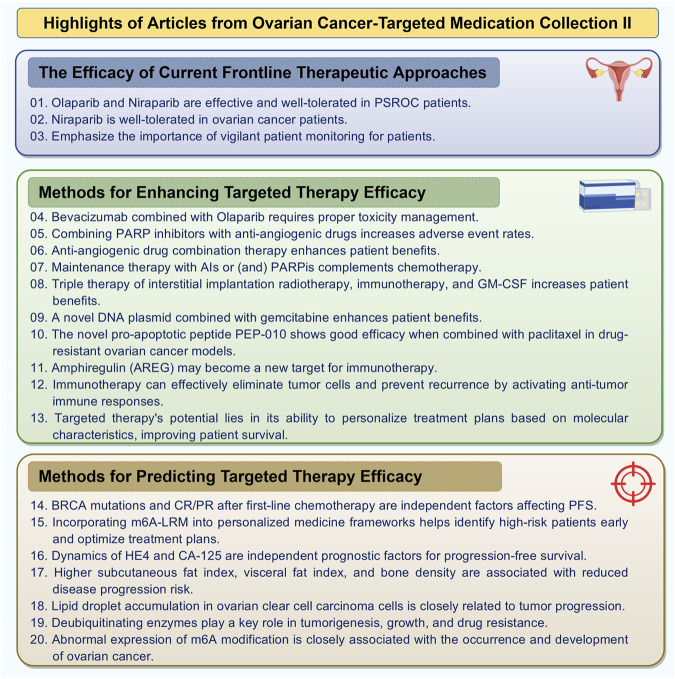
Highlights of articles from ovarian cancer-targeted medication collection (By FigDraw).

This Research Topic encompasses pharmacological topics, including immune-targeted therapy, prognostic biomarkers, and single-cell sequencing analysis of the immune microenvironment in ovarian cancer (Figure 1). The following section provides a concise summary of the key highlights from the 20 articles featured in this Research Topic.

Data derived from clinical settings have affirmed the efficacy of current frontline therapeutic approaches.

1. In an observational study, Chen et al. scrutinized the efficacy and safety of poly (ADP-ribose) polymerase inhibitors (PARPi) as a maintenance treatment in 75 Chinese patients with platinum-sensitive recurrent ovarian cancer (PSROC). The outcomes demonstrate that both olaparib and niraparib are efficacious and exhibit a favorable tolerability profile in this cohort. They emphasize that their study underscores the value of real-world evidence in elucidating treatment efficacy and bolsters the clinical use of PARPi for PSROC patients.
2. The research spearheaded by Wang et al. delved into treatment-related adverse events (TRAE) in ovarian cancer patients undergoing niraparib treatment subsequent to platinum-based chemotherapy. The study’s revelations indicate a markedly reduced incidence of TRAE of any grade and grade ≥3 during niraparib administration compared to chemotherapy, with notable reductions in anemia and neutrophil count decrements. This suggests that niraparib is well-tolerated in this patient cohort, underscoring its clinical potential.
3. Sun and Liu’s meta-analysis on the efficacy and safety of PARP inhibitor maintenance therapy for ovarian cancer indicates that PARP inhibitors markedly enhance progression-free survival (PFS) and overall survival (OS) compared to placebo, while concurrently elevating the risk of treatment-related adverse events. This finding accentuates the necessity for vigilant patient monitoring in clinical settings when employing PARP inhibitor maintenance therapy.

However, not all patient populations benefit from existing treatment regimens. The proposition and translational research of novel therapeutic modalities are imperative.

4. The expert synthesis of the safety profile of combining bevacizumab with olaparib as maintenance therapy for patients with newly diagnosed advanced ovarian cancer, based on the PAOLA-1 trial data, indicates that while the combination is deemed safe, there is a notable rate of treatment discontinuations due to adverse events. Romero et al. stress the importance of adept toxicity management to enhance patient quality of life and maximize treatment efficacy.
5. The review and meta-analysis, comparing the efficacy and safety of PARP inhibitors in combination with antiangiogenic agents in ovarian cancer treatment, reveals that combined therapy significantly improves PFS but also increases the incidence of adverse events. Wei et al. note that while combined therapy offers a clear advantage in PFS, its impact on OS remains uncertain.
6. The meta-analysis assesses the efficacy and safety of anti-angiogenic drug monotherapy and combination therapy in ovarian cancer, indicating that combination therapy markedly improves PFS and objective response rate (ORR), while monotherapy does not yield significant survival benefits. Xie and Zhou highlight the critical importance of adverse event monitoring in clinical practice.
7. Hao et al.’s bibliometric analysis, which explores the research progress in the treatment of recurrent ovarian cancer (ROC), shows a consistent increase in ROC treatment literature in recent years, with significant contributions from the United States and Italy. The analysis identifies research hotspots focused on PARP inhibitors and anti-angiogenic agents, indicating the growing importance of these novel therapies in ROC management.
8. A case report investigates a novel triple therapy approach for a patient with oligometastatic platinum-resistant ovarian cancer, combining interstitial implantation radiotherapy, immunotherapy, and granulocyte-macrophage colony-stimulating factor (GM-CSF). The patient showed a partial response to the treatment, with sustained benefits for over 6 months. Qin et al. suggest that this combination therapy may offer additional treatment options for patients with poor prognoses under conventional therapies.
9. The randomized controlled trial evaluates the clinical efficacy and safety of ELENAGEN, a novel DNA plasmid encoding p62/SQSTM1, in combination with gemcitabine for patients with platinum-resistant ovarian cancer. The results indicate that the ELENAGEN group achieved a median PFS of 7.2 months compared to 2.8 months in the gemcitabine-only group. Krasny et al. suggest that ELENAGEN may be effective, particularly in patients with poor prognostic factors, highlighting its potential as a new therapeutic option.
10. Lacroix et al. demonstrate that PEP-010, a first-in-class pro-apoptotic peptide, shows promising efficacy in both monotherapy and in combination with paclitaxel against resistant ovarian adenocarcinoma cell models. The study reveals that PEP-010 effectively induces apoptosis and significantly reduces the IC50 of paclitaxel, suggesting its potential application value in ovarian cancer treatment. This highlights the importance of exploring novel therapeutic strategies in combating drug resistance.
11. One study investigates how increased exposure to amphiregulin (AREG) affects the tumor immune microenvironment in high-grade serous ovarian cancer. The results indicate that increased AREG promotes immune evasion and tumor cell growth. Ebott et al. suggest that AREG may serve as a novel target for immunotherapy, highlighting its potential role in modulating the immune landscape of ovarian tumors.
12. Massariol’s study encapsulates recent advancements in immunotherapy for ovarian cancer, with a focus on strategies such as cancer vaccines, CAR-T cell therapy, and immune checkpoint inhibitors (Massariol Pimenta et al.). The researchers emphasize that despite the challenges, the potential of immunotherapy in treating ovarian cancer is substantial. They noted, “By activating anti-tumor immune responses, immunotherapy can effectively eliminate tumor cells and prevent recurrence.”
13. The narrative review discusses advancements in targeted treatments for ovarian cancer, including anti-angiogenic agents, PARP inhibitors, and immune checkpoint inhibitors (Satora et al.). The article underscores that while existing therapies delay recurrence, there is an urgent need for new strategies to enhance outcomes. The authors highlight that “the potential of targeted therapies lies in their ability to personalize treatment plans based on molecular characteristics, thereby enhancing patient survival rates.”

Regarding the early prediction of the efficacy of targeted therapy for ovarian cancer, several studies have provided commendable attempts and strategies.

14. One real-world study evaluates the use of PARPi as first-line maintenance therapy in newly diagnosed ovarian cancer patients at a major center in China. The study identifies BRCA mutation status and achieving complete or partial response after first-line chemotherapy as independent factors associated with prolonged PFS. As stated by Chen et al., these findings contribute valuable insights into the effectiveness of PARPi in clinical practice for ovarian cancer patients.
15. Ye’s study identified six N6-methyladenosine (m6A) effector-related long non-coding RNAs (lncRNAs) through machine learning and constructed a risk prediction model for serous ovarian carcinoma (Ye et al.). The findings indicate that high-risk patients have poorer prognoses and greater sensitivity to immunotherapy. As the researchers noted, “Incorporating the m6A-LRM into personalized medicine frameworks may help identify high-risk patients early and optimize treatment strategies.”
16. The META4 clinical trial investigates the prognostic value of HE4 and CA-125 kinetics in patients with recurrent epithelial ovarian carcinoma undergoing chemotherapy. The study finds that baseline concentrations of both biomarkers, as well as their nadir levels and time to nadir, are significant predictors of PFS. As highlighted by Fabbro et al., monitoring HE4 and CA-125 levels could enhance patient management and treatment decision-making in recurrent ovarian cancer.
17. Guo’s study examines the prognostic value of body composition and inflammation markers in patients with epithelial ovarian cancer treated with Olaparib. The findings reveal that higher subcutaneous adipose tissue index, visceral adipose tissue index, and bone mineral density are associated with a reduced risk of disease progression. As Guo et al. emphasize, these indicators could provide crucial references for personalized treatment strategies.
18. Koizume et al. propose that lipid droplets may serve as critical factors linking the biological backgrounds of ovarian clear cell carcinoma (OCCC) and clear cell renal cell carcinoma (ccRCC). The research highlights that lipid metabolism in OCCC cells remains underexplored, while lipid droplet accumulation in ccRCC cells is closely associated with tumor progression. This finding opens new avenues for potential therapeutic strategies in OCCC, underscoring the significance of lipid droplets in cancer research.
19. Qiu et al. conducted a bibliometric analysis to systematically review the progress of deubiquitinases (DUBs) in ovarian cancer research. The study finds a steady increase in literature related to DUBs since 1996, with significant contributions from China, the United States, and the United Kingdom. Keyword analysis reveals that DUBs play critical roles in tumor initiation, growth, and resistance, suggesting that future research should focus on their potential as therapeutic targets.
20. Alam’s study investigates the role of N6-methyladenosine (m6A) modification in ovarian cancer, emphasizing its significance in cancer progression, drug resistance, and therapeutic prospects. The findings indicate that aberrant expression of m6A modifications is closely associated with the onset and development of ovarian cancer, potentially serving as a novel prognostic marker. As Alam and Giri highlight, the regulatory mechanisms of m6A modifications offer new insights for personalized treatment strategies.

In summary, this Research Topic has provided us with profound insights into the development of targeted therapies for ovarian cancer (OC) and has furnished solid evidence to enhance their efficacy and reduce toxicity. Despite the histological characteristics of the ovarian tissue microenvironment and the “cold tumor” nature of OC, research on targeted therapies, particularly immunotherapy, still faces challenges. However, progress will eventually be made, even though the journey may be long.

Overall, this Research Topic has painted a comprehensive picture of the current state and future perspectives of targeted therapies for ovarian cancer. It has not only highlighted the significant advancements in understanding the complex molecular mechanisms of OC but also underscored the tangible progress in treatment strategies. The evidence accumulated from these studies helps us refine treatment approaches, emphasizing the need for personalized medicine and the potential of combination therapies to improve patient outcomes.

